# The Functions of Cytokines in the Cardiac Immunopathogenesis of Chagas Disease

**DOI:** 10.3390/pathogens13100870

**Published:** 2024-10-03

**Authors:** Mariana Citlalli de Alba-Alvarado, Margarita Cabrera-Bravo, Edgar Zenteno, Paz María Salazar-Schetino, Martha Irene Bucio-Torres

**Affiliations:** 1 Departamento de Microbiología y Parasitología, Facultad de Medicina, Universidad Nacional Autónoma de México, Ciudad México 04510, Mexico; imay@unam.mx (M.C.-B.); pazmar@unam.mx (P.M.S.-S.); 2 Departamento de Bioquímica, Facultad de Medicina, Universidad Nacional Autónoma de México, Ciudad de México 04510, Mexico; ezenteno@unam.mx

**Keywords:** *Trypanosoma cruzi*, Chagas disease, cardiopathy, cytokines, Th1, Th17

## Abstract

Chagas disease is a complex zoonosis. Clinically, it presents in two distinct phases, acute and chronic. The ability of patients to respond to *Trypanosoma cruzi* infection depends on the balance between inflammatory and anti-inflammatory responses, in which cytokines play a key regulatory role. In this review, we discuss the role of cytokines in regulating the host response and as mediators of cardiac injury by inducing profibrotic alterations. The importance of characterizing cytokine profiles as biomarkers of the evolution of cardiac damage in *T.-cruzi*-infected individuals is also emphasized.

## 1. Introduction

Chagas disease is a complex zoonosis involving interactions between vector species and wild, domestic, and peridomestic mammals, with several known modes of transmission [1]. In humans, the disease presents with different clinical manifestations as it progresses. The causative agent is the flagellated protozoan *Trypanosoma cruzi*, which is naturally transmitted by the excretions of triatomines, a subfamily of hematophagous hemipterans; this intracellular parasite was described by Dr. Carlos Chagas in Brazil in 1909 [2]. The vector colonizes homes in rural and suburban areas, keeping the infection close to population centers. In these environments, squirrels, opossums, voles, and armadillos participate in transmission as mammalian reservoirs [3].

Chagas disease is characterized by two clinical phases: acute and chronic. In the acute phase, the signs and symptoms are related to the portal of entry of the parasite, including the Romaña–Mazza sign (unilateral bipalpebral edema) or an inoculation chagoma (subcutaneous nodular lesion), often accompanied by nonspecific systemic manifestations (5% of cases), such as fever, adenitis, myalgia, arthralgia, hepatosplenomegaly, and anemia [1]. Atypical or less-common manifestations in this phase include acute cardiomyopathy, meningoencephalitis [4], lymphochagoma, (not reported in Mexico) [5], and skin rash [6] particularly in immunocompromised patients. The chronic phase of Chagas disease can present asymptomatically or symptomatically. The asymptomatic phase, characterized by low or no parasitemia, usually lasts between 10 and 20 years, and is diagnosed by serological methods. In the symptomatic phase, the main effects involve the heart, the digestive system, and the nervous system [7,8].

Approximately 27% of patients develop progressive heart failure evidenced by an incomplete block of the right bundle branch, left anterior hemiblock, and ventricular extrasystoles [8,9]. Echocardiography reveals apical aneurysms in 8.5–55% of cases, intramural thrombi in the left ventricle, and ventricular dysfunction with a reduced ejection fraction [10,11]. In this phase, visceral manifestations are infrequently observed, including a megacolon and megaesophagus with alterations of the nervous system [12,13]. On the other hand, atypical complications such as pericarditis and sudden death [14] can occur in both phases of the disease.

The immune response in the vertebrate host depends on the clinical phase of infection, the presence of cytokine-producing immune cells, and other host genetic factors. With respect to the parasite, factors such as virulence, inoculum size, and the recurrence of reinfection, which is common in areas of high endemicity, are important [15].

Controversy remains regarding the variables involved in the clinical evolution following infection, with 30% of patients progressing to the chronic symptomatic phase, while 70% remain asymptomatic [16]; it can be inferred that the expression of cytokine profiles determined by host genetics, as well as the response to parasite antigens, interact to contribute to this progression.

Cytokine profiles, as key regulators of the immune response, play an important role in controlling parasitism and regulating the inflammatory process in Chagas disease [15,16,17,18]. Therefore, this review aims to provide a context for their role in the clinical evolution of the disease as it progresses to chronicity.

## 2. Role of Cytokines in the Acute Phase of Chagas Disease

Despite the parasite’s mechanisms of evading the host immune response, Toll-like receptors (TLRs) are capable of recognizing specific parasite antigens on the *T. cruzi* membrane. These receptors are expressed on phagocytes, macrophages, and cells of the innate response; among these antigens, glycosylphosphatidylinositol (GPI), recognized by TLR2 and TLR6 [19], and glycosylinositol phospholipids (GIPLs), recognized by TLR4, stand out. On the other hand, oligonucleotide sequences with non-methylated CpG motifs of *T. cruzi* DNA are recognized by TLR9, which is highly expressed on dendritic cells [20].

TLR activation initiates a MyD88-dependent signaling cascade that activates transcription factors and promotes the expression of proinflammatory cytokines such as IL-12 and subsequently interferon gamma (IFN-*γ*). It has been shown experimentally that MyD88-knockout mice are susceptible to acute *T. cruzi* infection [21]. Activation of these receptors also induces various transcription factors that promote cytokine expression, thereby stimulating both innate and adaptive immune responses.

After primary infection, the immune response mainly involves the activation of immune cells such as neutrophils, eosinophils, and phagocytic cells. During the acute phase of infection, neutrophils, one of the most abundant innate cell types, are recruited to the site of infection through the action of IL-17 [22]. In response to parasitic antigens, neutrophils produce extracellular traps (NETS) and proinflammatory cytokines such as tumor necrosis factor-alpha (TNF-*α*) and IL-1 [23].

During the acute phase, the production of cytokines with proliferative properties, such as the hematopoietic growth factor (IL-3) and IL-2, has also been reported. Such production is extremely important in the acute phase because it stimulates the development of NK cells. NK cells are crucial in the innate response against the parasite because they produce IFN-*γ* and TNF-*α*, and these cytokines will lead to the elimination of *T. cruzi* by inducing the activation of macrophages in the early phase of infection [24].

TNF-*α* promotes inflammation in infected individuals, as well as apoptosis of *T.-cruzi*-infected cells. This cytokine, together with IL-6 [25], increases iNOS activation in macrophages, in addition to increasing the expression of chemokines to induce migration of other immune cells to the site of infection, in synergy with IFN-*γ* [26,27,28]. Macrophages are phagocytic cells that can polarize into different subsets depending on microenvironmental stimuli, parasite antigens, and signaling through PPAR*γ* [28]. In the acute phase of infection, M1 macrophages play an inflammatory role, stimulating the expression of molecules such as iNOS, NADPH, NO, and ROS, and increasing the expression of MHC class II, CD86, AP-1, and NF-*κ*B. They also produce cytokines such as TNF-*α*, IL-6, IL-12, and IL-1*β* [29,30,31]. This response can lead to parasite destruction, but it can also promote inflammation and tissue damage driven by the response to parasite antigens.

On the other hand, the M2 macrophage subset is associated with tissue remodeling and parasitic phagocytosis. M2 macrophages secrete Th2-type cytokines such as IL-10 and transforming growth factor beta (TGF-*β*) and express molecules such as CD36, CD163, mannose receptor (MR), AMPK, Arg1, PPARs, and STAT6 [32,33,34], which are associated with an immunoregulatory response and extracellular matrix remodeling.

**Figure 1:**
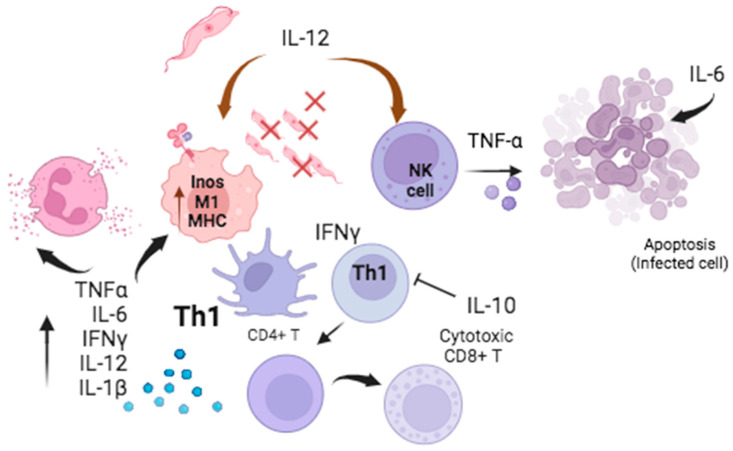
Major cytokines in the acute response. IFN-*γ* activates phagocytic cells and iNOS, reducing parasitemia and increasing MHC expression, which optimize the antigen presentation by APCs. TNF-*α* in synergy with IL-6 promotes apoptosis of infected cells and activates neutrophils and eosinophils. IL-10 inhibits the early IFN-*γ* response. Created with Bio-Render.com.

The role of monocytes and macrophages is crucial at the onset of infection. Activation of these cells is mediated by proinflammatory Th1-type cytokines that are important for phagocytosis. The major cytokines involved in this acute-phase response are IL-12, IFN-*γ*, and TNF-α [35,36] (Figure 1). IL-12 appears to be the cytokine initiator and promoter of the acute-phase response against *T. cruzi* [37]; IL-12 is also required for the production of mediators for the transcription and generation of IFN-*γ* and TNF-*α*. Some authors have indicated that when the function of this cytokine is blocked by anti-IL-12 antibodies, the survival of blood trypomastigotes during the acute phase is increased because there is no initial proinflammatory response [37]. Although the important role of IL-12 is known, the master control for the development of the Th1-type response depends on the presence of interferon gamma (IFN-*γ*) [20,38]. During this phase, phagocytic cells or M1 macrophages are activated by IFN-*γ*, which stimulates the inducible nitric oxide synthase (iNOS) pathway in macrophages; this activation is necessary to lyse parasites in the blood during the acute phase [39], along with macrophage activation and an increase in MHC expression to promote the function of antigen-presenting cells (APC) [17,40] (Figure 1).

## 3. Role of Cytokines in Chronic Heart Disease

In the acute phase of Chagas disease, the proinflammatory immune response should be regulated to control tissular and circulating parasitism. In some patients, this response is dysregulated, promoting the establishment of a persistent inflammatory process.

IFN-*γ* is considered the backbone for an effective Th1 response at the onset of infection; however, high levels of this cytokine during the chronic phase can affect the myocardium in some individuals. Its expression is associated with susceptibility to damage during this phase by inducing a cytotoxic and proinflammatory cellular response [41,42].

TNF-*α* is thought to induce the formation of reactive oxygen intermediates and promote the deposition of the extracellular matrix in the late phase of the disease, leading to cardiomyocyte necrosis and apoptosis [43,44]. In addition, it promotes the expression and high levels of IL-6, which contribute to the development of thrombotic processes by increasing endothelial platelet adhesion and stimulating platelet production [45].

IL-1*β* induces the production of peptides that deregulate osmosis in myocardial cells, producing the myocardial hypertrophy commonly described in these patients. Finally, IL-2 has been implicated in the abnormal activation of T lymphocytes in other dilated cardiomyopathies [46]; its involvement in chronic Chagas disease has been reported [47,48,49].

In the chronic phase, an increase in the circulating concentrations of several chemokines has been demonstrated, due to the increased expression of IL-8. This has implications in the induction of myocardial ischemia, since it is a platelet-attracting factor and favors the formation of platelet thrombi at the microvascular level, causing ischemia and tissue necrosis, important elements in the pathogenesis of lesions in Chagas disease [48,50]. In addition, there are reports of the involvement in this stage of the disease of some chemokine receptors, such as CXCL10 and CXCL9, which are expressed in the cardiac tissue and have been associated with a large infiltration of CD8+ T cells in the myocardium in a murine model [51]. The major cytokines involved in the pathogenesis of lesions in the chronic phase of Chagas disease are listed in Table 1.

**Figure 2:**
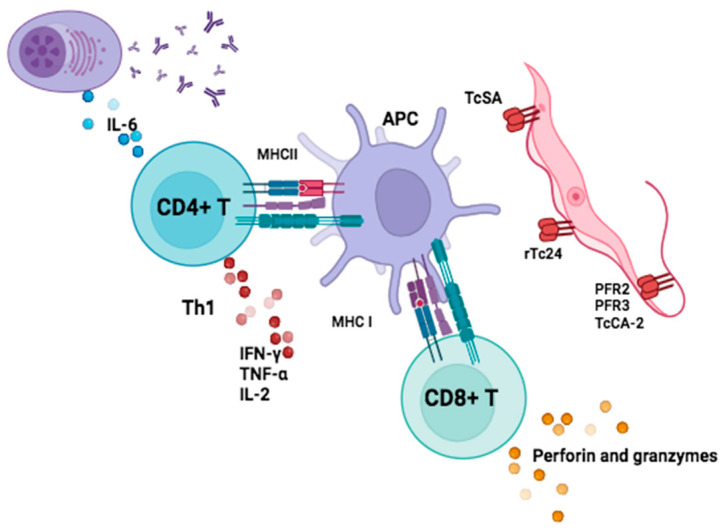
Cytokine induction by *T. cruzi* antigens. The TSA-1 and Tc24 antigens induce IFN-*γ* production by T lymphocytes. TcSA antigen induces IFN-*γ*, TNF*α*, and IL-2 production by CD4+ T and maintains the Th1 response of granzyme- and perforin-producing CD8+ T. Created with BioRender.com.

Although studies investigating cytokine production following *T. cruzi* antigen presentation are scarce, some authors have specifically described surface, kinetoplast, and flagellar antigens. All of these antigens are inducers of the Th1 response, activating plasma cells via IL-6 and the effector mechanisms of CD8 T cells. It has been reported that the *r*TSA-1 and *r*Tc24 antigens are inducers of IFN-*γ* production in circulating T lymphocytes from patients with Chagas heart disease [64]; similarly, the Y-strain TcSA antigen leads to the production of IFN-*γ*, TNF*α*, and IL-2 by T CD4 lymphocytes, maintaining the Th1-type response described above (Figure 2). However, some authors agree that there is a loss of functionality of both CD4+ and CD8+ T lymphocytes. This may be important in the progression to chronicity as it maintains T lymphocytes in a senescent phenotype [65,66].

## 4. Cytokines with Profibrotic Effect

**Figure 3:**
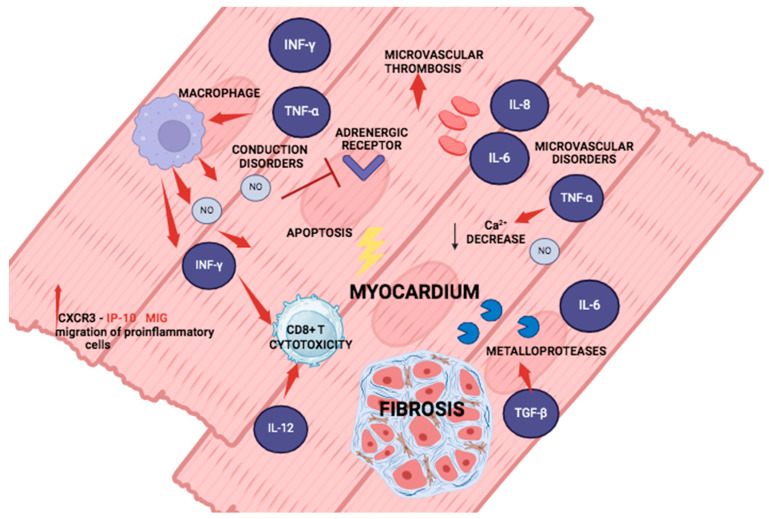
Fibrotic cytokines and chemokines in cardiac tissue during the chronic phase. Continuous Th1 cytokine production maintains NO in macrophages, promoting apoptosis. IL-12 maintains and IFN-*γ* stimulates CD8+ T cytotoxicity. CXCR3 and its ligands maintain the proinflammatory cell migration. IL-8 promotes thrombus formation and tissue ischemia. IL-6 and TNF-*α* inhibit the calcium pathway, affecting myocardial contractility. TGF-*β* induces metalloprotease production, resulting in extracellular matrix degradation, collagen production, and fibrosis. Created with BioRender.com.

Cardiac fibrosis is an important process in the progression of the chronic phase of Chagas disease. TGF-*β* exerts master control in regulating the formation and degradation of the extracellular matrix, in addition to inducing the expression of essential components such as fibronectin, laminin, and collagen, and in the generation of fibrosis [67]. Other cytokines are also considered profibrotic in the myocardium, although they have not been fully identified in Chagas disease, including TNF-*α*, endothelin, platelet-derived growth factor (PDGF), and basic fibroblast growth factor (BFGF). In addition, cardiac fibroblasts have been described as acquiring a proinflammatory phenotype characterized by the secretion of cytokines and chemokines such as IL-8, IL-1*β*, CCL2, eotaxin, and TNF-*α* with extracellular matrix-degrading properties [68,69] (Figure 3)

In Chagas disease, myocardial fibrosis is involved in the genesis of tachyarrhythmias (ventricular tachycardia or fibrillation) because fibrosis can block the cardiac stimulus and reduce conduction velocity. In addition, collagen septa between the muscle bundles may interfere with the conduction of the electrical impulse, which is expressed on ECG tracing as a right bundle branch block or hemiblock of the left anterior fascicle of the bundle of His [70,71].

In a study of patients with Chagas heart disease, septal hypertrophy and left ventricular posterior wall hypertrophy were found. These lesions correlate with increased levels of proinflammatory and profibrotic cytokines [62,63]. Other authors have suggested that the persistent inflammation may be the consequence of a delayed hypersensitivity immune response, with activation of memory T cells and recruitment of mononuclear cells to the site of inflammation [72].

Several pathogenic mechanisms have been described as promoting tissue damage in the chronic phase of the disease, including inflammation, conduction and microvascular dysfunction, which collectively trigger the process of cardiac fibrosis [71]. Proinflammatory cytokines are also involved in these mechanisms; an important one, TNF-*α*, together with IFN-*γ*, induces the activation of the iNOS pathway in macrophages, which by producing nitric oxide, induce cardiac cell apoptosis and inhibit beta-adrenergic receptors [73]. In a cascade of events amplified by the continued production of IL-6 and TNF-*α*, the calcium pathway is inhibited, further impairing myocardial contractility and leading to cardiac conduction abnormalities [74] (Figure 3). In addition, the expression of IFN-*γ* and IL-12 can maintain the activation of CD8+ T lymphocytes, which produce granzyme b and perforin; both cellular factors contribute to necrosis and cytotoxic tissue damage, which can be repaired by the production of collagen [75]. On the other hand, cytokines such as IL-6 and IL-8 modify the vascular endothelium and contribute to the formation of platelet-derived microthrombi [76,77], which alter the perfusion of cardiac tissue, causing ischemia and tissue necrosis. Together with TGF-*β*, ischemia induces the production of metalloproteases for the degradation of the extracellular matrix, resulting in the formation of collagen and ultimately fibrosis [78] (Figure 3).

## 5. Cytokines and Chemokines in Resistance and Susceptibility to *Trypanosoma Cruzi* Infection

Several authors emphasize the role of cytokines in Chagas disease because of their protective role during the acute phase (Table 2). A key function of cytokines and chemokines in controlling the parasite is to stimulate the functions of innate cells. Meanwhile, other authors suggest that these same cytokines, when deregulated or overexpressed during the chronic phase, may contribute to host susceptibility to the disease (Table 3).

Chemokine receptors (CXCR), such as CXCR3, promote the specific chemotaxis of CD8+ T cells to control infected cells [79]; however, ligands of these receptors, such as CXCL10 (IP-10), CXCL11, and CXCL9 (MIG), are produced by antigen-presenting cells in infected tissues, favoring the development of myocarditis with high levels of CD8+ T lymphocyte populations [26]; This chemokine receptor (CXCR3) continues to be studied and has been defined as an important mediator of the cytotoxic response [79]; on the other hand, CCR5 has been found to be overexpressed with a lower expression of CXCR4 in patients with mild heart disease compared to normal individuals, suggesting that these chemokines are involved in the development of early forms of Chagas cardiomyopathy [80,81]. Finally, resistance and susceptibility to infection or disease have been associated with cytokine and chemokine expression (Table 2 and Table 3).

**Table 2:** Cytokines and chemokines reported to induce protection in Chagas disease.

Cytokine	Protection	Ref.
IL-12	High levels in asymptomatic patients infected with *T. cruzi*.	[38] Michailowsky et al., 2001
IFN-*γ*	IFN-γ production is induced in patients on drug treatment (nifurtimox).	[41] Bahia et al., 1998
IFN-*γ* and TNF-*α*	In vitro stimulation of macrophages with IFN-*γ* reduces the number of amastigotes in the acute phase.	[18] Cardoni et al., 1999
IL-17-A	Detected in an acute-phase mouse model. Involved in parasite control.	[62] da Matta Guedes et al., 2010 [80] Miyazaki et al., 2010
IL-6	It contributes to mounting the response to the parasite, as well as to host resistance to *T. cruzi* infection during the acute phase.	[82] Gao et al., 2002
IL-27	In patients with Chagas disease without severe cardiopathy, its production has shown a tolerogenic effect.	[83] Natale et al., 2021
CCL3 and CCL5	In a mouse model, they are involved in mucosal protection and activation of the B-cell defense.	[84] Sullivan et al., 2011

**Table 3:** Cytokines and chemokines reported as susceptibility inducers of CHD. CHD: chronic heart disease.

Cytokine	Susceptibility	Ref.
IL-1, IL-6, IL-15, IFN-*γ*, TNF-*α*	Elevated levels in patients with CHD.	[40] Gomes et al., 2003
IL-10	Low levels in patients with CHD. By inhibiting macrophage proliferation in the acute phase, it mediates susceptibility to *T. cruzi* infection.	[85] Reed et al., 1994
TNF-*α*	Elevated levels in patients with dilated cardiomyopathy and in chronic, asymptomatic, and symptomatic patients.	[52] Ferreira et al., 2003
IL-1*β*	Elevated levels in patients with CHD.	[46] Petersen and Burleigh, 2003
IL-17	High levels associated with a significant increase in myocardial destruction. Elevated circulating levels in younger patients with CHD.	[62] da Matta Guedes et al., 2010 [63] De alba et al., 2018
IL-13	In combination with arginase-1, it promotes susceptibility to *T. cruzi* infection.	[86] Abad et al., 2018
IL-15	Found in the clinical cardiac form of the disease.	[40] Gomes et al., 2003
TGF-*β* and IL-10	Stimulation of macrophage cultures with high levels of both cytokines inhibits the trypanocidal effect of IFN-*γ*.	[18] Cardoni et al., 1999
Surrogate proteins for TGF-*β*, MMP-2, MMP-9, and fibronectin	Responsible for tissue fibrosis in pathological cardiac remodeling in CHD.	[87] Medeiros et al., 2019 [88] Bautista-López et al., 2013 [89] Fares et al., 2013 [67] Baron et al., 2022
CXCL9 (MIG), CXCL10, CCL2 CCR5, MCP-1CCXCR3	Increased levels of these chemokines in the heart tissue of patients with CHD. They are associated with increased IFN-*γ* levels. Necessary for recruitment of inflammatory cells.	[84] Sullivan et al., 2011

## 6. Host Genetics and Cytokine Expression

Host genetics is another key factor in cytokine expression and may explain the variation in cytokine levels between patients as well as susceptibility to heart disease in the chronic phase [90].Some authors are studying cytokine and chemokine gene polymorphisms to determine the possible genetic basis of cytokine dysregulation associated with various diseasesThe search for repeated microsatellite polymorphisms and single-nucleotide polymorphisms has been proposed. The former are used in linkage studies in nuclear family samples, and the latter are usually used in case-control association studies in population samples. Several authors have proposed the following alleles as inducers of susceptibility and resistance in Chagas disease (Table 4 and Table 5).

Interestingly, several authors have expressed interest in identifying single-nucleotide polymorphisms (SNPs) in cytokine genes that could be used as prognostic markers and to identify groups of cases prone to developing severe heart disease with Chagas disease. However, there is a consensus that solid genetic biomarkers are lacking due to the plasticity of the immune response and the inflammatory profile of each patient. These are not only determined by genetic factors, but also depend on the biology of the parasite and its virulence, as well as the biology of the host itself.

## 7. Parasite Genetics

It has been hypothesized that genetic variability in discrete typing units (DTUs) within the *T. cruzi* taxon would promote different cytokine profiles in the host, as the genetic pattern of the parasite influences the clinical picture of the disease. However, Poveda, in 2014, did not find a differential cytokine profile, although they reported greater inflammation in patients infected with TcII strains compared to those infected with TcI, which could be due to pleiotropic and redundant functions of the circulating cytokines [100]. Finally, it is noteworthy that the cytokine profile is proinflammatory for all DTUs, which ultimately leads to the same clinical and pathological inflammatory signs in the heart, gastrointestinal tract, and nervous system, regardless of the DTU.

## 8. Balance of Proinflammatory and Anti-inflammatory Cytokines

**Figure 4:**
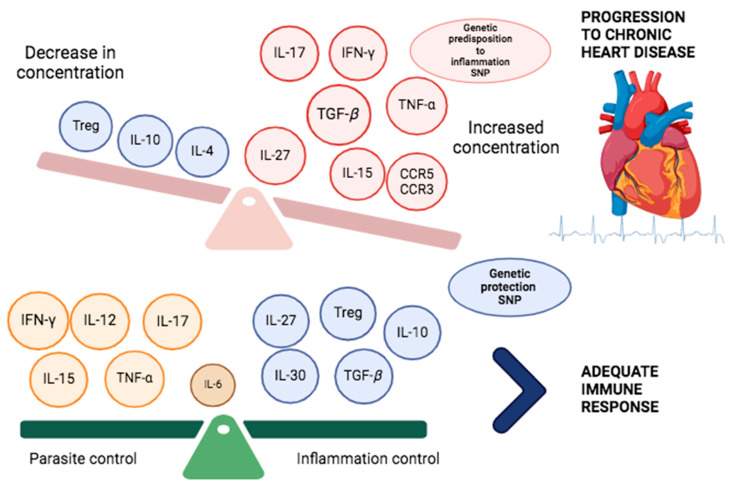
Balance between pro- and anti-inflammatory cytokines. At the top, there is sustained expression of proinflammatory/profibrotic cytokines (red) and their genes, predisposing to chronic disease. Initially, in the acute phase, proinflammatory cytokines are expressed to control parasitemia (yellow); subsequently, regulatory anti-inflammatory cytokines (blue) and their genes prevent and control inflammation. Created with BioRender.com.

Finally, it should be considered that a favorable outcome for the vertebrate is more likely if an adequate balance is established between the proinflammatory mediators involved in the antiparasitic Th1 response (IL-1, IL-6, IFN-*γ*, and TNF-*α*), which are expressed in the acute phase and subsequently regulated by anti-inflammatory cytokines (IL-10) [101]. In this regard, IL-10 not only inhibits the Th1 response, but also controls the responses of other cells (T and B lymphocytes); however, an excessive decrease in the levels of anti-inflammatory mediators (IL-10) and the deregulation of cytokines such as IFN-*γ* lead to a proinflammatory response with the continued presence of activated CD4+ and CD8+ T lymphocyte populations, which contributes to the progression of chronic disease [102] (see Figure 4).

Some cytokines may be at the center of the immune balance; among the most studied are IL-27, IL-17, and IL-6.

IL-27, generally produced by antigen-presenting cells, contains IL-30 as a subunit. Its main function is to regulate T and B lymphocytes, as both cells express a specific receptor for this cytokine. It has been shown experimentally that the administration of IL-27 improves the response of Th1 cells, both monofunctional and polyfunctional, in individuals without heart disease, along with an increase in the expression of IL-10 [103]. Its production has also been reported in other studies in patients with Chagas disease who do not have severe heart disease, suggesting that IL-27 production has a tolerogenic effect in these cases. As mentioned above, several authors have reported a marked exhaustion of the T-cell response in the chronic phase of Chagas disease [65,66]. In this regard, Natale (2021) suggests that this is due to the dysregulation between IL-7 and IL-27, and their receptors, which promotes the state of exhaustion of the T-cell response in the late phases of Chagas disease [83,103,104].

The function of IL-17 depends on the pro- or anti-inflammatory context in which it is expressed. There is evidence that it controls the Th1-type response or recruits IL-10-producing neutrophils, which favors host survival during acute infection by regulating an exacerbated immune response [22]. The levels of this cytokine have also been found to be elevated in patients with maternal–fetal infection associated with low parasitemia. Thus, IL-17 has been considered a protective cytokine during the acute phase [80]; however, like other proinflammatory cytokines, its uncontrolled overexpression is associated with a chronic inflammatory process in tissues [105,106]. Other studies suggest that IL-17 may contribute to pathogenicity when associated with Th1-type proinflammatory cytokines [62,107]; this response is even associated with chronic inflammatory autoimmune diseases [108]. Like IL-17, IL-6 can act with proinflammatory or anti-inflammatory function [109]. IL-6 signaling pathways are relevant in cardiomyocytes and macrophages involved in the anti-parasite response; however, most investigators support the hypothesis that this cytokine is important in inflammation and lesion pathogenesis in the chronic disease and has little effect on anti-inflammatory regulatory processes in Chagas disease [55,63,64,73,75].

The role of IL-10 and the tolerogenic dendritic cells that produce this cytokine, which plays a role in the regulation of inflammation by reducing proinflammatory Th1 profiles, which in turn modulates fibrosis, is currently under investigation. It has also been observed that IL-10, in combination with drugs such as fenofibrate, reduces the inflammatory cellular infiltrate and polarizes macrophages toward the M2 phenotype, favoring proper tissue remodeling [110].

There have been few studies on the involvement of TGF-*β*- and IL-10-producing regulatory T cells (Tregs), which may play an important role in regulating inflammation late in the acute phase and preventing clinical progression to the chronic phase [111] (Figure 4).

## 9. Conclusions

The immune response of the vertebrate host and the balance between proinflammatory and regulatory responses are key determinants of the clinical course of Chagas disease.

Th1- and Th17-type responses are required in the acute phase of the disease for parasite control by IFN-*γ* and IL-17. Later, their regulation by cytokines such as IL-10, IL-30, and IL-27 is equally important. However, the persistent expression of proinflammatory and profibrotic cytokines induces the progression of tissue lesions characteristic of cardiopathy in Chagas disease.

This work highlights the importance of studying these cytokines in situ, as shown in Figure 3, because most studies focus on the systemic response in the serum of infected patients. Thus, these cytokines can be considered as biomarkers of lesion development and clinical progression. On the other hand, they can be used to directly determine in tissues whether the inflammatory process in patients with Chagas disease is under control.

As an adjunct to specific antiparasitic therapies, cytokine levels may be useful as biomarkers of a cure or treatment failure, or to develop antiparasitic therapies in combination with drugs proven effective in modulating the inflammatory response to provide new alternatives in the treatment of Chagas-disease-related cardiopathies.

## 10. Perspective

Further therapeutic studies in patients at different clinical stages of the disease are needed to study the modulation of cytokine and chemokine activity that may favor the host response by controlling the excessive production of proinflammatory and profibrotic mediators involved in the chronic pathology of Chagas disease.

## Figures and Tables

**Table 1:** Major cytokines and chemokines involved in the chronic phase of Chagas disease (CHD). MO^a^, monocytes; MA^b^, macrophages; CE^c^, endothelial cells; NE^d^, neutrophils; CD4+ ^e^T, CD4+ lymphocytes; NK^f^, NK cells; CD^g^, dendritic cells; Th2, Th2-type response; Th1, Th1 lymphocytes; O^h^, other cell types.

Cytokine	Secreted by	Effect	Ref.
TNF-*α*	Th1, MA^b^, MO^a^, N^d^, LT^f^, NE^d^ and NK^f^	Elevated levels of TNF-*α*, are associated with left ventricular dysfunction in patients with CHD.	[52] Ferreira et al., 2003
		Plasma concentrations of TNF-α are higher in chronic patients compared to healthy controls.	[53] Rocha et al., 2012
IL-12	MA^b^, CD^g^, Th1	Elevated levels of IL-12 have been implicated in the pathophysiology of CHD in humans.	[54] Pereira et al., 2018
IL-6	MA^b^, CE^c^, and Th2	IL-6 levels increase with the progression of heart failure in Chagas disease.	[55] López et al., 2006
IL-2	Th1, CD4+ ^e^T	Patients with CHD have decreased peripheral IL-2 levels.	[49] Briceño et al., 1996
TGF-*β*	Th2, MA^b^, O^h^	Cardiac involvement is associated with increased activity of the TGF-*β* signaling pathway and expression of its key components.	[56] Ferreira et al., 2022
		Inhibition of TGF-*β* signaling reverses *T.-cruzi*-induced fibrosis and hypertrophy in chronic disease.	[57] Ferrão, et al., 2018
IFN-*γ*	Th1, NK^f^	Patients with CHD have increased levels of IFN-*γ* upon infection.	[41] Abel et al., 2001
		The response to infection, along with IFN-*γ* production, can depress energy metabolism (myocardial ATP), affecting myocardial contractility, electrical conduction, and heart rate.	[58] Ferreira et al., 2014
		In patients with CHD, several lines of evidence point to the importance of IFN-γ in the pathogenesis of myocarditis and heart failure.	[59] Chevillard et al., 2018
IL-10	MA^b^, MO^a^, Th2, O^h^	Low IL-10 expression is associated with cardiac dysfunction in terms of left ventricular ejection fraction.	[60] Costa et al., 1999
		In patients with cardiomyopathy, elevated serum IL-10 levels have been described in asymptomatic patients without cardiomegaly.	[61] Vasconcelos et al., 2015
IL-17	CD4+ ^e^T	In the pathogenesis of myocarditis, IL-17 production is associated with decreased IL-12 expression.	[62] da Matta et al., 2010
		Patients with severe cardiac manifestations have a Th17 profile with significantly higher levels of IL-17 and IL-6 in addition to IFN-γ and IL-2.	[63] De Alba-Alvarado et al., 2018

**Table 4:** Major cytokine and chemokine genes and alleles reported to be protective against the development of cardiopathy in Chagas disease. * Gene, ** single-nucleotide polymorphism, SNP.

Genetic Protection			
Cytokine	Gene/Polymorphism	Function	Ref.
IL-12	IL12B * (rs2546893] ** (rs919766] **	High expression in asymptomatic or acutely infected patients with *T. cruzi*. Significant in IFN-*γ* production and Th1 cell differentiation.	[35] Frade-Barros et al., 2020
IL-10	IL10 * (rs1800896] **	It has been associated with reduced disease severity and upregulation of the anti-inflammatory immune response.	[24] Sathler-Avelar et al., 2003
CCR5	*−59029AG* (rs1799987) **	It has been associated with lower CCR5 expression, which may confer protection against the development of severe cardiac symptoms.	[91] Fernández-Mestre et al., 2004
CXCL10, CCL5, CXCL9	CXCL9 * (rs10336 CC **), CXCL10 * (rs 3921 GG **) CCR5 * (rs1799988CC **)	These chemokines are considered master regulators of inflammatory cell migration in the myocardium. A reduction in their expression is associated with less-severe myocarditis.	[92] Nogueira et al., 2012

**Table 5:** Major cytokine and chemokine genes and alleles reported in susceptibility to the development of CHD. * Gene, ** single-nucleotide polymorphism, SNP. CHD: chronic heart disease.

Genetic Susceptibility			
Cytokine	Gene/Polymorphism	Function	Ref.
TNF family, sTNFR1 and sTNFR2	sTNFR1 (rs767455) ** sTNFR2(rs1061624) ** TNFA−1031TT * TNFA−308GG *, TNFa2 * TNFa7 *, TNFa8 * TNFb2 *, TNFb4 * TNFd5 *, TNFd7* TNFe2 *	They modulate TNF-*α* signaling. They are associated with systemic inflammation and progression of CHD.	[93] Alvarado-Arnez et al., 2018
IFN-γ	*IFNG +874T/A*	It has been associated with susceptibility to CHD in patients in Colombia.	[94] Torres et al.,2010
IL-12	IL12B *	It has been associated with susceptibility to CHD.	[95] Zafra et al., 2007
IL-1B	IL1B +5810G * *IL1B * −31 +395 CT **	Its elevated expression has been associated with an excessive inflammatory response that contributes to tissue damage and progression in CHD.	[96] Flórez et al., 2011 [97] Cruz-Robles et al., 2009
	*IL * *−1RN.4CC*		
IL-4	*IL4 −590C/T* [rs2243250) **	The polymorphism in the promoter region of the gene enhances the Th2 response, which limits the inflammatory process in the acute phase and favors parasite persistence.	[96] Flórez et al., 2011
IL-17	IL17A * (rs2275913) **	It has been significantly associated with an increased risk of developing CHD.	[98] Strauss et al., 2020
TGF-β	TGFB1*	It confers susceptibility in patients with CHD in Peru.	[99] Calzada et al., 2009
CCR5	CCR5 −2554T * (rs1799987) **	It affects CCR5 expression, which is associated with immune cell migration to the site of infection and the inflammatory response in CHD.	[91] Fernández-Mestre et al., 2004
	*−2733G* (rs1799988) **		

## References

[1] National Autonomous University of Mexico (2006). Manual para el Diagnóstico de la Infección por Trypanosoma cruzi.

[2] Chagas C. (1909). Nova tripanozomiaze humana: Estudos sobre a morfolojia e o ciclo evolutivo do Schizotrypanum cruzi n. gen., n. sp., ajente etiolojico de nova entidade morbida do homem. Mem. Inst. Oswaldo Cruz.

[3] Ruiz-Piña H.A., Cruz-Reyes A. (2002). The Opossum Didelphis Virginiana as a Synanthropic Reservoir of *Trypanosoma cruzi* in Dzidzilché, Yucatán, México. Mem. Inst. Oswaldo Cruz.

[4] Del Valle Pugliese Uliarte D., Durante L.A.T., Rivas D.M., Villegas V.G., Maestre K.S., De Soto A.J.B. (2014). Acute Miocarditis and Meningoencephalitis Caused by *Trypanosoma cruzi* in an Hiv-Seropositive Patient. Rev. Cuba. Med. Trop..

[5] Quintal Avilés R., Zavala Velázquez J., Pinzón Cantarell J. (1976). La enfermedad de Chagas en el estado de Yucatán México (informe de transmisores). Salud Publica Mex..

[6] Ferraresso M.G., Torre A.C., Piva M.M.M., Barcan L. (2018). Chagas disease reactivation: Cutaneous manifestations in a transplanted patient. An. Bras. Dermatol..

[7] Teixeira A.R., Nascimento R.J., Sturm N.R. (2006). Evolution and pathology in Chagas disease—A Review. Mem. Inst. Oswaldo Cruz.

[8] Salazar-Schettino P.M., Bucio-Torres M.I., Cabrera-Bravo M., Citlalli De Alba-Alvarado M., Rocío Castillo-Saldaña D., Zenteno-Galindo E.A., Rojo-Medina J., Angélica Fernández-Santos N., Perera-Salazar G. (2016). Enfermedad de Chagas en México. Rev. Fac. Med..

[9] Sánchez Y.S., Talavera R.V., Bravo C.B., Huerta L.V., Benzaquen E.C., Díaz F.D., Echegaray J.B., Altamirano J.G., Oviedo L.R. (2007). Estudio comparativo de alteraciones electrocardiográficas, frecuencia cardiaca y presión arterial entre seropositivos y seronegativos para *Trypanosoma cruzi* en el valle de Vítor, Arequipa-Perú. Acta Médica Peru..

[10] Vinajá D., Aché A. (2012). Alteraciones Electrocardiográficas en pacientes con Enfermedad de Chagas. Hospital José Rangel de Villa de Cura. 1998–2008. Rev. Inst. Nac. Hig. Rafael Rangel.

[11] Barbosa M.M., Nunes M.C.P. (2012). Risk stratification in chagas disease. Rev. Esp. Cardiol..

[12] Köberle F. (1968). Chagas’ Disease and Chagas’ Syndromes: The Pathology of American Trypanosomiasis. Adv. Parasitol.

[13] Useche Y., Pérez A.R., de Meis J., Bonomo A., Savino W. (2022). Central Nervous System Commitment in Chagas Disease. Front. Immunol..

[14] Nunes M.C.P., Beaton A., Acquatella H., Bern C., Bolger A.F., Echeverría L.E., Dutra W.O., Gascon J., Morillo C.A., Oliveira-Filho J. (2018). Chagas Cardiomyopathy: An Update of Current Clinical Knowledge and Management: A Scientific Statement From the American Heart Association. Circulation.

[15] De Fuentes-Vicente J.A., Vidal-López D.G., Flores-Villegas A.L., Moreno-Rodríguez A., De Alba-Alvarado M.C., Salazar-Schettino P.M., Rodríguez-López M.H., Gutiérrez-Cabrera A.E. (2019). *Trypanosoma cruzi*: A review of biological and methodological factors in Mexican strains. Acta Trop..

[16] Moncayo A. (1999). Progress towards interruption of transmission of Chagas disease. Mem. Inst. Oswaldo Cruz.

[17] Abbas A.K., Lichtman A.H., Pillai S. (2018). Inmunología Celular y Molecular.

[18] Cardoni R.L., Antunez M.I., Abrami A.A. (1999). Respuesta TH1 en la infeccion experimental con *Trypanosoma cruzi*. Medicina.

[19] Campos M.A.S., Almeida I.C., Takeuchi O., Akira S., Valente E.P., Procópio D.O., Travassos L.R., Smith J.A., Golenbock D.T., Gazzinelli R.T. (2001). Activation of Toll-Like Receptor-2 by Glycosylphosphatidylinositol Anchors from a Protozoan Parasite. J. Immunol..

[20] Bartholomeu D.C., Ropert C., Melo M.B., Parroche P., Junqueira C.F., Teixeira S.M.R., Sirois C., Kasperkovitz P., Knetter C.F., Lien E. (2008). Recruitment and Endo-Lysosomal Activation of TLR9 in Dendritic Cells Infected with *Trypanosoma cruzi*. J. Immunol..

[21] Campos M.A., Closel M., Valente E.P., Cardoso J.E., Akira S., Alvarez-Leite J.I., Ropert C., Gazzinelli R.T. (2004). Impaired Production of Proinflammatory Cytokines and Host Resistance to Acute Infection with *Trypanosoma cruzi* in Mice Lacking Functional Myeloid Differentiation Factor 88. J. Immunol..

[22] Boari T.T., Vesely A., Bermejo M.C., Ramello D.A., Montes M.C. (2012). IL-17RA Signaling Reduces Inflammation and Mortality during *Trypanosoma cruzi* Infection by Recruiting Suppressive IL-10-Producing Neutrophils. PLoS Pathog..

[23] Futosi K., Fodor S., Mócsai A. (2013). Neutrophil cell surface receptors and their intracellular signal transduction pathways. Int. Immunopharmacol..

[24] Sathler-Avelar R., Lemos E.M., Reis D.D., Medrano-Mercado N., Araujo-Jorge T.C., Antas P.R.Z., Corrêa-Oliveira R., Teixeira-Carvalho A., Elói-Santos S.M., Favato D. (2003). Phenotypic Features of Peripheral Blood Leucocytes during Early Stages of Human Infection with *Trypanosoma cruzi*. Scand. J. Immunol..

[25] Laucella S.A., Rottenberg M.E., Titto E.H. (1996). Papel de las citoquininas en la resistencia y patología durante la infección con *Trypanosoma cruzi*. Rev. Argent. Microbiol..

[26] Dos Santos P.V.A., Roffê E., Santiago H.C., Torres R.A., Marino A.P.M.P., Paiva C.N., Silva A.A., Gazzinelli R.T., Lannes-Vieira J. (2001). Prevalence of CD8^+^αβ T cells in *Trypanosoma cruzi*-elicited myocarditis is associated with acquisition of CD62LLowLFA-1HighVLA-4High activation phenotype and expression of IFN-γ-inducible adhesion and chemoattractant molecules. Microbes Infect..

[27] Antúnez M.I., Cardoni R.L. (2000). IL-12 and IFN-γ production, and NK cell activity, in acute and chronic experimental *Trypanosoma cruzi* infections. Immunol. Lett..

[28] Lawrence T., Natoli G. (2011). Transcriptional regulation of macrophage polarization: Enabling diversity with identity. Nat. Rev. Immunol..

[29] Hovsepian E., Mirkin G.A., Penas F., Manzano A., Bartrons R., Goren N.B. (2011). Modulation of inflammatory response and parasitism by 15-Deoxy-Δ12,14 prostaglandin J2 in *Trypanosoma cruzi*-infected cardiomyocytes. Int. J. Parasitol..

[30] Díaz-Gandarilla J.A., Osorio-Trujillo C., Hernández-Ramírez V.I., Talamás-Rohana P. (2013). PPAR activation induces m1 macrophage polarization via cPLACOX-2 inhibition, activating ros production against Leishmania Mexicana. Biomed. Res. Int..

[31] Martins R.M., Alves R.M., Macedo S., Yoshida N. (2011). Starvation and rapamycin differentially regulate host cell lysosome exocytosis and invasion by *Trypanosoma cruzi* metacyclic forms. Cell. Microbiol..

[32] Sag D., Carling D., Stout R.D., Suttles J. (2008). Adenosine 5′-Monophosphate-Activated Protein Kinase Promotes Macrophage Polarization to an Anti-Inflammatory Functional Phenotype. J. Immunol..

[33] Basso B. (2013). Modulation of immune response in experimental Chagas disease. World J. Exp. Med..

[34] Zanluqui N.G. (2015). Macrophage Polarization in Chagas Disease. J. Clin. Cell. Immunol..

[35] Farage Frade-Barros A., Maria Ianni B., Cabantous S., Wide Pissetti C., Saba B., Tzu Lin-Wang H., Buck P., Antonio Marin-Neto J., Schmidt A., Dias F. (2020). Polymorphisms in Genes Affecting Interferon-γ Production and Th1 T Cell Differentiation Are Associated with Progression to Chagas Disease Cardiomyopathy. Front. Immunol..

[36] Teixeira D.E., Benchimol M., Crepaldi P.H., de Souza W. (2012). Interactive Multimedia to Teach the Life Cycle of *Trypanosoma cruzi*, the Causative Agent of Chagas Disease. PLoS Negl. Trop. Dis..

[37] Aliberti J.C.S., Cardoso M.A.G., Martins G.A., Gazzinelli R.T., Vieira L.Q., Silva J.S. (1996). Interleukin-12 mediates resistance to *Trypanosoma cruzi* in mice and is produced by murine macrophages in response to live trypomastigotes. Infect. Immun..

[38] Michailowsky V., Silva N.M., Rocha C.D., Vieira L.Q., Lannes-Vieira J., Gazzinelli R.T. (2001). Pivotal role of interleukin-12 and interferon-γ axis in controlling tissue parasitism and inflammation in the heart and central nervous system during *Trypanosoma cruzi* infection. Am. J. Pathol..

[39] Guiñazú N., Pellegrini A., Carrera-Silva E.A., Aoki M.P., Cabanillas A.M., Gìronés N., Fresno M., Cano R., Gea S. (2007). Immunisation with a major *Trypanosoma cruzi* antigen promotes pro-inflammatory cytokines, nitric oxide production and increases TLR2 expression. Int. J. Parasitol..

[40] Gomes J.A.S., Bahia-Oliveira L.M.G., Rocha M.O.C., Martins-Filho O.A., Gazzinelli G., Correa-Oliveira R. (2003). Evidence that development of severe cardiomyopathy in human Chagas’ disease is due to a Th1-specific immune response. Infect. Immun..

[41] Bahia-Oliveira L.M.G., Gomes J.A.S., Rocha M.O.C., Moreira M.C.V., Lemos E.M., Luz Z.M.P., Pereira M.E.S., Coffman R.L., Dias J.C.P., Cançado J.R. (1998). IFN-γ in human Chagas’ disease: Protection or pathology?. Braz. J. Med. Biol. Res..

[42] Abel L.C.J., Rizzo L.V., Ianni B., Albuquerque F., Bacal F., Carrara D., Bocchi E.A., Teixeira H.C., Mady C., Kalil J. (2001). Chronic Chagas’ disease cardiomyopathy patients display an increased IFN-γ response to *Trypanosoma cruzi* infection. J. Autoimmun..

[43] Lima E.S., Andrade Z.A., Andrade S.G. (2001). TNF-α is expressed at sites of parasite and tissue destruction in the spleen of mice acutely infected with *Trypanosoma cruzi*. Int. J. Exp. Pathol..

[44] Engel D., Peshock R., Armstong R.C., Sivasubramanian N., Mann D.L. (2004). Cardiac myocyte apoptosis provokes adverse cardiac remodeling in transgenic mice with targeted TNF overexpression. Am. J. Physiol.—Heart Circ. Physiol..

[45] Antunes D., Marins-Dos-Santos A., Ramos M.T., Mascarenhas B.A.S., De Carvalho Moreira C.J., Farias-De-Oliveira D.A., Savino W., Monteiro R.Q., De Meis J. (2019). Oral Route Driven Acute *Trypanosoma cruzi* Infection Unravels an IL-6 Dependent Hemostatic Derangement. Front. Immunol..

[46] Petersen C.A., Burleigh B.A. (2003). Role for interleukin-1β in *Trypanosoma cruzi*-induced cardiomyocyte hypertrophy. Infect. Immun..

[47] Salazar-Schettino P.M., Cabrera-Bravo M., Vazquez-Antona C., Zenteno E., De Alba-Alvarado M., Torres Gutierrez E., Gomez Y.G., Perera-Salazar M.G., de la Torre G.G., Bucio-Torres M.I. (2016). Chagas disease in Mexico: Report of 14 cases of chagasic cardiomyopathy in children. Tohoku J. Exp. Med..

[48] Adamopoulos S., Parissis J.T., Kremastinos D.T. (2001). A glossary of circulating cytokines in chronic heart failure. Eur. J. Heart Fail..

[49] Briceño L., Mosca W. (1996). Defective production of interleukin 2 in patients with Chagas’ disease. Purified IL-2 augments in vitro response in patients with chagasic cardiomyopathy. Mem. Inst. Oswaldo Cruz.

[50] Marin-Neto J.A., Cunha-Neto E., Maciel B.C., Simões M.V. (2007). Pathogenesis of chronic Chagas heart disease. Circulation.

[51] Talvani A., Ribeiro C.S., Aliberti J.C., Michailowsky V., Santos P.V., Murta S.M., Romanha A.J., Almeida I.C., Farber J., Lannes-Vieira J. (2000). Kinetics of cytokine gene expression in experimental chagasic cardiomyopathy: Tissue parasitism and endogenous IFN-γ as important determinants of chemokine mRNA expression during infection with *Trypanosoma cruzi*. Microbes Infect..

[52] Ferreira R.C., Ianni B.M., Abel L.C., Buck P., Mady C., Kalil J., Cunha-Neto E. (2003). Increased plasma levels of tumor necrosis factor-alpha in asymptomatic/“indeterminate” and Chagas disease cardiomyopathy patients. Mem. Inst. Oswaldo Cruz.

[53] Rodrigues D.B.R., Dos Reis M.A., Romano A., Pereira S.A.D.L., Teixeira V.D.P.A., Tostes Junior S., Rodrigues V. (2012). In situ expression of regulatory cytokines by heart inflammatory cells in chagas’ disease patients with heart failure. J. Immunol. Res..

[54] de Sena Pereira N., Queiroga T.B.D., Nunes D.F., de Mesquita Andrade C., Nascimento M.S.L., Do-Valle-Matta M.A., da Câmara A.C.J., da Cunha Galvão L.M., Guedes P.M.M., Chiari E. (2018). Innate immune receptors over expression correlate with chronic chagasic cardiomyopathy and digestive damage in patients. PLoS Negl. Trop. Dis..

[55] López L., Arai K., Giménez E., Jiménez M., Pascuzo C., Rodríguez-Bonfante C., Bonfante-Cabarcas R. (2006). Las concentraciones séricas de interleucina-6 y proteína C reactiva se incrementan a medida que la enfermedad de Chagas evoluciona hacia el deterioro de la función cardíaca. Rev. Española Cardiol..

[56] Ferreira R.R., Waghabi M.C., Bailly S., Feige J.J., Hasslocher-Moreno A.M., Saraiva R.M., Araujo-Jorge T.C. (2022). The Search for Biomarkers and Treatments in Chagas Disease: Insights From TGF-Beta Studies and Immunogenetics. Front. Cell. Infect. Microbiol..

[57] Ferrão P.M., Nisimura L.M., Moreira O.C., Land M.G., Pereira M.C., de Mendonça-Lima L., Araujo-Jorge T.C., Waghabi M.C., Garzoni L.R. (2018). Inhibition of TGF-β pathway reverts extracellular matrix remodeling in *T. cruzi*-infected cardiac spheroids. Exp. Cell Res..

[58] Ferreira L.R.P. (2014). Interferon-γ and other inflammatory mediators in cardiomyocyte signaling during Chagas disease cardiomyopathy. World J. Cardiol..

[59] Chevillard C., Nunes J.P.S., Frade A.F., Almeida R.R., Pandey R.P., Nascimento M.S., Kalil J., Cunha-Neto E. (2018). Disease Tolerance and Pathogen Resistance Genes May Underlie *Trypanosoma cruzi* Persistence and Differential Progression to Chagas Disease Cardiomyopathy. Front. Immunol..

[60] Costa G.C., Rocha M.O.D.C., Moreira P.R., Menezes C.A.S., Silva M.R., Gollob K.J., Dutra W.O. (2009). Functional IL-10 gene polymorphism is associated with Chagas disease cardiomyopathy. J. Infect. Dis..

[61] Vasconcelos R.H.T., Azevedo E.A.N., Diniz G.T.N., Cavalcanti M.G.A.M., de Oliveira W., de Morais C.N.L., Gomes Y.M. (2015). Interleukin-10 and tumour necrosis factor-alpha serum levels in chronic Chagas disease patients. Parasite Immunol..

[62] Guedes P.M.D.M., Gutierrez F.R.S., Maia F.L., Milanezi C.M., Silva G.K., Pavanelli W.R., Silva J.S. (2010). IL-17 produced during *Trypanosoma cruzi* infection plays a central role in regulating parasite-induced myocarditis. PLoS Negl. Trop. Dis..

[63] De Alba-Alvarado M., Salazar-Schettino P.M., Jiménez-Álvarez L., Cabrera-Bravo M., García-Sancho C., Zenteno E., Vazquez-Antona C., Cruz-Lagunas A., Zúñiga J., Bucio-Torres M.I. (2018). Th-17 cytokines are associated with severity of *Trypanosoma cruzi* chronic infection in pediatric patients from endemic areas of Mexico. Acta Trop..

[64] Villanueva-Lizama L.E., Cruz-Chan J.V., Aguilar-Cetina A.d.C., Herrera-Sanchez L.F., Rodriguez-Perez J.M., Rosado-Vallado M.E., Ramirez-Sierra M.J., Ortega-Lopez J., Jones K., Hotez P. (2018). *Trypanosoma cruzi* vaccine candidate antigens Tc24 and TSA-1 recall memory immune response associated with HLA-A and -B supertypes in Chagasic chronic patients from Mexico. PLoS Negl. Trop. Dis..

[65] Albareda M.C., Olivera G.C., Laucella S.A., Alvarez M.G., Fernandez E.R., Lococo B., Viotti R., Tarleton R.L., Postan M. (2009). Chronic Human Infection with Trypanosoma Cruzi Drives CD4+ T Cells to Immune Senescence. J Immunol.

[66] Mateus J., Guerrero P., Lasso P., Cuervo C., González J.M., Puerta C.J., Cuéllar A. (2019). An animal model of acute and chronic chagas disease with the reticulotropic Y strain of *Trypanosoma cruzi* that depicts the multifunctionality and dysfunctionality of T cells. Front. Immunol..

[67] Araújo-Jorge T.C., Waghabi M.C., Soeiro M.d.N.C., Keramidas M., Bailly S., Feige J.J. (2008). Pivotal role for TGF-beta in infectious heart disease: The case of *Trypanosoma cruzi* infection and consequent Chagasic myocardiopathy. Cytokine Growth Factor Rev..

[68] Baron M.A., Ferreira L.R.P., Teixeira P.C., Moretti A.I.S., Santos R.H.B., Frade A.F., Kuramoto A., Debbas V., Benvenuti L.A., Gaiotto F.A. (2022). Matrix Metalloproteinase 2 and 9 Enzymatic Activities are Selectively Increased in the Myocardium of Chronic Chagas Disease Cardiomyopathy Patients: Role of TIMPs. Front. Cell. Infect. Microbiol..

[69] Borthwick L.A., Wynn T.A., Fisher A.J. (2013). Cytokine Mediated Tissue Fibrosis. Biochim. Biophys. Acta.

[70] Dolber P.C., Spach M.S. (1987). Thin Collagenous Septa in Cardiac Muscle. Anat. Rec..

[71] De Alba-Alvarado M.C., Torres-Gutiérrez E., Reynoso-Ducoing O.A., Zenteno-Galindo E., Cabrera-Bravo M., Guevara-Gómez Y., Salazar-Schettino P.M., Rivera-Fernández N., Bucio-Torres M.I. (2023). Immunopathological Mechanisms Underlying Cardiac Damage in Chagas Disease. Pathogens.

[72] Abrahamsohn I.A., Blotta M.H.S.L., Curotto M.A. (1981). Enhancement of delayed-type hypersensitivity to *Trypanosoma cruzi* in mice treated with Mycobacterium bovis BCG and cyclophosphamide. Infect. Immun..

[73] Satoh M., Nakamura M., Tamura G., Makita S., Segawa I., Tashiro A., Satodate R., Hiramori K. (1997). Inducible Nitric Oxide Synthase and Tumor Necrosis Factor-Alpha in Myocardium in Human Dilated Cardiomyopathy. J. Am. Coll. Cardiol..

[74] Kinugawa K.I., Takahashi T., Kohmoto O., Yao A., Aoyagi T., Momomura S.I., Hirata Y., Serizawa T. (1994). Nitric oxide-mediated effects of interleukin-6 on [Ca2+]i and cell contraction in cultured chick ventricular myocytes. Circ. Res..

[75] Silverio J.C., De-Oliveira-Pinto L.M., Da Silva A.A., De Oliveira G.M., Lannes-Vieira J. (2010). Perforin-expressing cytotoxic cells contribute to chronic cardiomyopathy in *Trypanosoma cruzi* infection. Int. J. Exp. Pathol..

[76] De Jonge E., Friederich P.W., Vlasuk G.P., Rote W.E., Vroom M.B., Levi M., Van der Poll T. (2003). Activation of coagulation by administration of recombinant factor VIIa elicits interleukin 6 (IL-6) and IL-8 release in healthy human subjects. Clin. Vaccine Immunol..

[77] Pérez-Campos Mayoral L., Hernández-Huerta M.T., Papy-García D., Barritault D., Zenteno E., Sánchez Navarro L.M., Pérez-Campos Mayoral E., Matias Cervantes C.A., Martínez Cruz M., Mayoral Andrade G. (2021). Immunothrombotic Dysregulation in Chagas Disease and COVID-19: A Comparative Study of Anticoagulation. Mol. Cell. Biochem..

[78] Kobayashi T., Kim H.J., Liu X., Sugiura H., Kohyama T., Fang Q., Wen F.Q., Abe S., Wang X., Atkinson J.J. (2014). Matrix metalloproteinase-9 activates TGF-β and stimulates fibroblast contraction of collagen gels. Am. J. Physiol.-Lung Cell. Mol. Physiol..

[79] Pontes Ferreira C., Cariste L.M., Ferri Moraschi B., Ferrarini Zanetti B., Won Han S., Araki Ribeiro D., Vieira Machado A., Lannes-Vieira J., Gazzinelli R.T., Vasconcelos J.R.C. (2019). CXCR3 chemokine receptor guides *Trypanosoma cruzi*-specific T-cells triggered by DNA/adenovirus ASP2 vaccine to heart tissue after challenge. PLoS Negl. Trop. Dis..

[80] Talvani A., Rocha M.O.C., Ribeiro A.L., Correa-Oliveira R., Teixeira M.M. (2004). Chemokine Receptor Expression on the Surface of Peripheral Blood Mononuclear Cells in Chagas Disease. J. Infect. Dis..

[81] Gomes J.A.S., Bahia-Oliveira L.M.G., Rocha M.O.C., Busek S.C.U., Tekeira M.M., Silva J.S., Correa-Oliveira R. (2005). Type 1 Chemokine Receptor Expression in Chagas’ Disease Correlates with Morbidity in Cardiac Patients. Infect Immun.

[82] Gao W., Pereira M.A. (2002). Interleukin-6 is required for parasite specific response and host resistance to Trypanosomacruzi. Int. J. Parasitol..

[83] Natale M.A., Minning T., Albareda M.C., Eiro M.D.C., Álvarez M.G., Lococo B., Cesar G., Bertocchi G., Elias M.J., Caputo M.B. (2021). Immune exhaustion in chronic Chagas disease: Pro-inflammatory and immunomodulatory action of IL-27 in vitro. PLoS Negl. Trop. Dis..

[84] Sullivan N.L., Eickhoff C.S., Zhang X., Giddings O.K., Lane T.E., Hoft D.F. (2011). Importance of the CCR5-CCL5 axis for mucosal *Trypanosoma cruzi* protection and B cell activation. J. Immunol..

[85] Reed S.G., Brownell C.E., Russo D.M., Silva J.S., Grabstein K.H., Morrissey P.J. (1994). IL-10 mediates susceptibility to *Trypanosoma cruzi* infection. J. Immunol..

[86] Dar M.A., Hölscher C. (2018). Arginase-1 is responsible for IL-13-mediated susceptibility to *Trypanosoma cruzi* infection. Front. Immunol..

[87] Medeiros N.I., Gomes J.A.S., Fiuza J.A., Sousa G.R., Almeida E.F., Novaes R.O., Rocha V.L.S., Chaves A.T., Dutra W.O., Rocha M.O.C. (2019). MMP-2 and MMP-9 plasma levels are potential biomarkers for indeterminate and cardiac clinical forms progression in chronic Chagas disease. Sci. Rep..

[88] Bautista-López N.L., Morillo C.A., López-Jaramillo P., Quiroz R., Luengas C., Silva S.Y., Galipeau J., Lalu M.M., Schulz R. (2013). Matrix metalloproteinases 2 and 9 as diagnostic markers in the progression to Chagas cardiomyopathy. Am. Heart J..

[89] Fares R.C.G., Correa-Oliveira R., de Araújo F.F., Keesen T.S.L., Chaves A.T., Fiuza J.A., Ferreira K.S., Rocha M.O.C., Gomes J.A.S. (2013). Identification of phenotypic markers of B cells from patients with Chagas disease. Parasite Immunol..

[90] Gómez I., Thomas M.C., Palacios G., Egui A., Carrilero B., Simón M., Valladares B., Segovia M., Carmelo E., López M.C. (2021). Differential Expression of Immune Response Genes in Asymptomatic Chronic Chagas Disease Patients Versus Healthy Subjects. Front Cell Infect Microbiol.

[91] Fernández-Mestre M.T., Montagnani S., Layrisse Z. (2004). Is the CCR5-59029-G/G genotype a protective factor for cardiomyopathy in Chagas disease?. Hum. Immunol..

[92] Nogueira L.G., Santos R.H.B., Ianni B.M., Fiorelli A.I., Mairena E.C., Benvenuti L.A., Frade A., Donadi E., Dias F., Saba B. (2012). Myocardial Chemokine Expression and Intensity of Myocarditis in Chagas Cardiomyopathy Are Controlled by Polymorphisms in CXCL9 and CXCL10. PLoS Negl. Trop. Dis..

[93] Alvarado-Arnez L.E., Batista A.M., Alves S.M., Melo G., de Lorena V.M.B., Cardoso C.C., Pereira I.R., Carrazzone C., Pacheco A.G., Oliveira W. (2018). Single nucleotide polymorphisms of cytokine-related genes and association with clinical outcome in a Chagas disease case-control study from Brazil. Mem. Inst. Oswaldo Cruz.

[94] Torres O.A., Calzada J.E., Beraún Y., Morillo C.A., González A., González C.I., Martín J. (2010). Role of the IFNG +874T/A polymorphism in Chagas disease in a Colombian population. Infect. Genet. Evol..

[95] Zafra G., Morillo C., Martín J., González A., González C.I. (2007). Polymorphism in the 3′ UTR of the IL12B gene is associated with Chagas’ disease cardiomyopathy. Microbes Infect..

[96] Flórez O., Martín J., González Rugeles C.I. (2011). Interleukin 4, interleukin 4 receptor-α and interleukin 10 gene polymorphisms in Chagas disease. Parasite Immunol..

[97] Cruz-Robles D., Chvez-Gonzlez J.P., Cavazos-Quero M.M., Prez-Mndez O., Reyes P.A., Vargas-Alarcn G. (2009). Association between IL-1B and IL-1RN Gene Polymorphisms and Chagas’ Disease Development Susceptibility. Immunol. Invest..

[98] Strauss M., Palma-Vega M., Casares-Marfil D., Bosch-Nicolau P., Lo Presti M.S., Molina I., González C.I., Paglini P.A., Schijman A.G., Robello C. (2020). Genetic polymorphisms of IL17A associated with Chagas disease: Results from a meta-analysis in Latin American populations. Sci. Rep..

[99] Calzada J.E., Beraún Y., González C.I., Martín J. (2009). Transforming growth factor beta 1 (TGFβ1) gene polymorphisms and Chagas disease susceptibility in Peruvian and Colombian patients. Cytokine.

[100] Poveda C., Fresno M., Gironès N., Martins-Filho O.A., Ramírez J.D., Santi-Rocca J., Marin-Neto J.A., Morillo C.A., Rosas F., Guhl F. (2014). Cytokine Profiling in Chagas Disease: Towards Understanding the Association with Infecting *Trypanosoma cruzi* Discrete Typing Units (A BENEFIT TRIAL Sub-Study). PLoS ONE.

[101] Hovsepian E., Penas F., Siffo S., Mirkin G.A., Goren N.B. (2013). IL-10 Inhibits the NF-κB and ERK/MAPK-Mediated Production of Pro-Inflammatory Mediators by Up-Regulation of SOCS-3 in *Trypanosoma cruzi*-Infected Cardiomyocytes. PLoS ONE.

[102] de Souza Santos E., Silva D.K.C., Reis B.P.Z.C.D., Barreto B.C., Cardoso C.M.A., Santos R.R.D., Meira C.S., Soares M.B.P. (2021). Immunomodulation for the Treatment of Chronic Chagas Disease Cardiomyopathy: A New Approach to an Old Enemy. Front. Cell. Infect. Microbiol..

[103] Joseph W., Carl J., Bai X.-F. (2008). IL27: Its Roles in the Induction and Inhibition of Inflammation. Int. J. Clin. Exp. Pathol..

[104] Pérez-Antón E., Thomas M.C., Egui A., López M.C. (2019). T-Cell Exhaustion Process during Chronic Infection Caused by Intracellular Trypanosomatids. Ars Pharmaceutica.

[105] Kitada S., Kayama H., Okuzaki D., Koga R., Kobayashi M., Arima Y., Kumanogoh A., Murakami M., Ikawa M., Takeda K. (2017). BATF2 inhibits immunopathological Th17 responses by suppressing Il23a expression during *Trypanosoma cruzi* infection. J. Exp. Med..

[106] Albareda M.C., Perez-Mazliah D., Natale M.A., Castro-Eiro M., Alvarez M.G., Viotti R., Bertocchi G., Lococo B., Tarleton R.L., Laucella S.A. (2015). Perturbed T Cell IL-7 Receptor Signaling in Chronic Chagas Disease. J. Immunol..

[107] Magalhães L.M.D., Villani F.N.A., Nunes M.D.C.P., Gollob K.J., Rocha M.O.C., Dutra W.O. (2013). High Interleukin 17 Expression Is Correlated With Better Cardiac Function in Human Chagas Disease. J. Infect. Dis..

[108] Kim B.S., Park Y.J., Chung Y. (2016). Targeting IL-17 in autoimmunity and inflammation. Arch. Pharm. Res..

[109] Luo Y., Zheng S.G. (2016). Hall of fame among pro-inflammatory cytokines: Interleukin-6 gene and its transcriptional regulation mechanisms. Front. Immunol..

[110] Rada J., Donato M., Penas F.N., Alba Soto C., Cevey Á.C., Pieralisi A.V., Gelpi R., Mirkin G.A., Goren N.B. (2020). IL-10-Dependent and -Independent Mechanisms Are Involved in the Cardiac Pathology Modulation Mediated by Fenofibrate in an Experimental Model of Chagas Heart Disease. Front. Immunol..

[111] de Araújo F.F., Corrêa-Oliveira R., Rocha M.O.C., Chaves A.T., Fiuza J.A., Fares R.C.G., Ferreira K.S., Nunes M.C.P., Keesen T.S., Damasio M.P.S. (2012). Foxp3^+^CD25^high^ CD4^+^ regulatory T cells from indeterminate patients with Chagas disease can suppress the effector cells and cytokines and reveal altered correlations with disease severity. Immunobiology.

